# Effect of dietary *Eucommia ulmoides* oliver polysaccharide on immune function and meat quality of Songliao Black Pigs

**DOI:** 10.1038/s41598-024-64257-4

**Published:** 2024-06-17

**Authors:** Yang Liang, Zeyu Tang, Hao Wang, Meng Liu, Fanglin Zhao, Longsheng Wang, Yinbiao Meng, Lijun Jia

**Affiliations:** Engineering Research Center of North-East Cold Region Beef Cattle Science & Technology Innovation, Ministry of Education, Yanbian University, No.977 Park Road, Yanji, 133002 People’s Republic of China

**Keywords:** *Eucommia ulmoides polysaccharide*, SongLiao Black pigs, Immune function, Meat quality traits, Gene expression, Drug discovery, Molecular biology, Zoology

## Abstract

*Eucommia ulmoides* is a traditional Chinese herbal medicine, with pharmacological effects such as lowering blood pressure and enhancing immune function. The effects of dietary *Eucommia ulmoides* polysaccharide (EUP) on immune function and meat quality were studied in Songliao Black Pigs. Blood lymphocyte counts and percentage, concentrations of serum total protein and of albumin increased, whereas those of urea nitrogen and triglyceride decreased. White blood cell and lymphocyte counts, and serum IgA, IgE, IgG2 a and IFN-γ increased. Average daily weight gain, slaughter weight, lean meat rate and cooked meat rate increased, whereas pH_24_, feed-weight ratio, fat rate, yellowness (b^#^) and centrifugal dehydration rate decreased. Transcriptome sequencing of longissimus dorsi muscle detected 32 differentially expressed genes (DEGs), of which 26 were up-regulated and 6 down-regulated. A total of 19 genes were differentially expressed in the four groups, 18 of which were up-regulated. The DEGs included ADAMTS4, PER1, STAC, SERPINE1, FASN, THRSP, SP7 and KRT80 and the protein interaction network showed 20 up-regulated nodes, three down-regulated nodes and 14 DEGs. GO functional annotation and enrichment analysis showed that 34 items were significantly enriched, including transferase activity, actin binding, acetyl coenzyme A, acyl coenzyme A metabolism, adipose tissue development and acyl glycerol homeostasis. KEGG pathway analysis showed that the AMPK and PPAR signaling pathways were enriched. Dietary *Eucommia* polysaccharide enhanced immune function in Songliao Black Pigs, improved growth and carcass performance, increased the expression of genes related to meat quality traits and improved meat quality.

## Introduction

*Eucommia ulmoides* Oliver (*Eucommia*), also known as kapok, is a monophyletic deciduous tree^1^. *Eucommia* has a medicinal history of more than 2000 years in China, and is a commonly used Chinese medicinal material, containing a variety of bioactive substances^2^; the main components are lignans, iridoids, flavonoids, phenylpropanoids, terpenoids and polysaccharides^3,4^. The pharmacodynamic function and mechanism of *Eucommia* have received extensive attention. The pharmacodynamic functions of the different components are basically the same, but the mechanisms of action are different^5^. The main efficacies of *Eucommia* in humans are immune regulation, lowering blood pressure, controlling blood lipids, anti-diabetic, anti-oxidation, anti-aging, anti-fatigue, anti-bacterial, anti-inflammatory, anti-virus, anti-tumor, inhibition of osteoporosis, liver and kidney protection, sedation and prevention of miscarriage^6^.

Songliao Black pigs have black coats, a long middle body, strong limbs, strong reproductive performance, a high lean meat percentage and good meat quality, with the characteristics of high adaptability, rapid growth, low feed/meat ratio and strong stress resistance. Songliao Black pigs are bred by crossing the northeast native pig with the landrace and Duroc pigs; they have strong cold resistance and can live normally at – 30 °C, so they are suitable for farming in Northeast China^7^. There have been many reports on the application of *Eucommia* as a dietary additive in white pigs, but few studies on black pigs^8^. Dietary *Eucommia* polysaccharide improves growth performance, enhances immunity, improves meat quality and other performance factors in white pigs, so this study determined the effects of *Eucommia* on Songliao Black pigs. The findings are of great significance for the environmentally-friendly production of safe and healthy food.

## Methods

### Ethics statement

All protocols and procedures involving animals were performed in accordance with the Regulations for the Administration of Affairs Concerning Experimental Animals (Ministry of Science and Technology, China) and were approved by the Animal Welfare Committee of China Yanbian University. During the experimental period, the animals were reared in the same environment, were fed the same diet and were humanely sacrificed. The study is reported in accordance with ARRIVE guidelines.

### Animals and experimental groups

Adult Songliao Black pigs were provided by Antu pig breeding farm (Antu, China). All animals were reared under the same conditions, with natural, uncontrolled temperature and light. The 40 pigs were randomly divided into four groups of 10, i.e., a control group and experimental groups I, II and III. The control group was fed a basic diet, based on corn-soybean meal and made according to the nutritional requirements for pigs in Nutrient Requirements of Swine Eleventh Revised Edition (2012, China). The composition was: corn 60%, rice bran 15%, soybean meal 12%, wheat bran 10%, fish meal 1%, vitamins 1%, mildew preservative 0.5% and sodium chloride 0.5%. Experimental groups I, II and III were fed diets supplemented with 1, 2, or 3% w/w *Eucommia ulmoides* polysaccharide, respectively. *Eucommia ulmoides* polysaccharide was supplied by Zhangjiajie Biotechnology Co., (Zhangjiajie, Hunan, China). 20% purity the powdered finished product. The monosaccharide compositions of EUP included Glu 58%, Gal 15%, Gum 11%, Rha 8%, Man 5% and Xyl 3%. The trial period was 30 days and each pig was fed separately.

### Sample collection

At the end of the 30 day trial period and after fasting for 12 h, venous blood (5 ml) was collected from all test animals in EDTA-K2 anticoagulation centrifuge tubes (Becton Dickinson, Franklin Lakes, NJ), centrifuged for 10 min at 600 × g and stored at – 20 °C for later analysis. The animals were slaughtered in a commercial abattoir and everything possible was done to minimize animal suffering during the study. The longissimus dorsi muscle from each animal was collected aseptically and as quickly as possible. Samples were frozen immediately and stored in liquid nitrogen until needed for RNA extraction. Samples for other assays were collected as described below, in the relevant method sections.

### Detection of routine blood and serum biochemical indices

The blood samples were analyzed on a GRT-6008 automatic blood analyzer (Glite Technology Co., Ltd., Jinan, China), to determine red blood cell, white blood cell, lymphocyte, monocyte and granulocyte counts; lymphocyte, monocyte and granulocyte percentages; hemoglobin concentration, average hemoglobin content, mean hemoglobin concentration; mean red blood cell volume, hematocrit, red blood cell distribution width SD, red blood cell distribution width CV; platelet hematocrit, mean platelet volume, platelet distribution width and large platelet ratio. The serum biochemical indices, total protein, albumin, globulin, urea nitrogen, triglyceride and glucose were determined by automatic dry biochemical analyzer (Fuji film imaging machine Co., Ltd., Suzhou, China).

### Detection of immunoglobulin and cytokine levels

Serum samples from the pigs in the control group and group II were analyzed by ELISA Kit (Langton Biotechnology Co., Ltd., Shanghai, China) to determine the serum immune indices, immunoglobulins IgG1, IgG2a, IgG2b, IgA and IgE, and cytokines IFN-γ, IL-4 and TNF-α, Standard curves were plotted according to the test kit manufacturer’s instructions and the immune indices were calculated according to the standard curve regression equation.

### Determination of growth performance and carcass performance

Three pigs were randomly selected from each test group. The pigs were weighed on an empty stomach at 8:00 a.m. on the first and last days of the trial, and the average daily weight gain was calculated. The feed intake of each pig was also recorded. The feed-to-weight ratio was calculated from the feed intake and average daily weight gain. With reference to the Measurement Methods of Pig Production^9^, the indices, pre-slaughter weight, carcass weight, eye muscle area, backfat thickness, skin weight, bone weight and skin thickness were measured, then the slaughter rate, lean meat rate and other related indices were calculated from the measurements.

### Determination of meat quality traits

After the slaughter of the selected pigs (previous section) and determination of growth/carcass performance, the longissimus dorsi and psoas major muscles of the left half-carcass of the same animals were quickly removed for meat quality determination, i.e., meat color, marbling, pH_24_, cooked meat rate, water loss rate, drip loss, tenderness value and intramuscular fat were determined according to the methods in the Chinese Code for Determination of Pig Muscle Quality (NY/T821-2004).

### Transcriptome sequencing

Three pigs were randomly selected from both the control group and group II; muscle tissue samples were collected, frozen in liquid nitrogen and sent to Beijing Annoroad Gene Technology Co., Ltd for transcriptome sequencing.Total RNA was extracted and according to the structure of mRNA 3′ end with polyA tail, the first strand of cDNA was synthesised using mRNA as template, then the second strand of cDNA was synthesised and the cDNA library was prepared by purification with QIAQuick PCR kit and end repair. Qubit 3.0 was used for preliminary quantification, the library was diluted to 1 ng/µl, the insert size of the library was detected using an Agilent 2100, and the effective concentration of the library was accurately quantified using Q-PCR with the Bio-RAD KIT iQ SYBR GREEN (the effective concentration of the library was > 10 nM).

### Data collection and sequence alignment

The raw sequencing read data were filtered to remove the sequences (reads) containing sequencing linkers, unknown bases with N ratios greater than 5%, or of low quality (number of bases with mass value Q ≤ 19 accounted for > 50% of the total bases), to obtain high-quality sequences (clean reads) meeting the requirements for subsequent analysis. The clean reads were compared with the pig reference genome (sus_scrofa. sscrofa11.1.90.chr:FTP/ftp.ensemble.org/ pub/release-90/fasta/sus_scrofa/DNA/sus_scrofa.sscrofa11.1).

### Analysis of differentially expressed genes (DEGs)

The number of reads for each gene was compared using the Python package HTSeq, the DEGs were identified using the R package DESeq and their expression was calculated by the FPKM algorithm from RNA-Seq. Significantly different DEGs were selected with the criteria: − log2 Fold change (FC) ≥ 1 and q < 0.05, and a volcano map, a cluster map and a Wayne map were drawn.

### Analysis of protein interaction network

According to the interaction relationships in the STRING protein interaction database, the target gene set was directly mapped to the protein interaction network. The network data file of protein interaction was directly imported into Cytoscape software, and the network was visually edited according to the gene attributes in the target gene set.

### Significant enrichment analysis of DEGs by GO function and KEGG pathway analyses

The DEGs obtained were compared with each entry in The GO database (http://www.geneontology.org/). After the P-value was corrected by the Benjamini method^10^, GO entries with Q < 0.05 were designated as significantly enriched entries. By comparing with the KEGG^11–13^ (Kyoto Encyclopedia of Genes and Genomes) database (http://wego.genomics.org.cn), the DEGs involved in signaling pathways, or metabolic pathways were analyzed.

### qPCR verification of gene expression

Primers were designed using oligo7.56 software, and the relative expression of five key genes, including CREB1, FASN, MLYCD, CPT1A and UBC, was quantified using fluorescence quantitative PCR.

CREB1-F: GTGTGTTACGTGGGGGAGAG; CREB1-R: GCATCTCCACTCTGCTGGTT. FASN-F: TCACCTACGAGGCCATTGTG; FASN-R: TCAGAACTGCTCACACCCAC.

MLYCD-F: GATCACCACGGCCATCTTCT; MLYCD-R: ACTCCTTCTGCAGCTCCTTC.

CPT1A-F: TGAGTGACCATTTGCCTGCT; CPT1A-R: CACGCCCGTGTCTTCTTTTG.

UBC-F: GAGAGCTGAATCCTTTGGGGAA; UBC-R: AGAAACGCCGAGAAGGGACT.

### Statistical analysis of data

Excel software was used to sort and analyze the test data, and the results were expressed as the mean ± standard deviation. Graph Pad Prism 5.0 software was used for independent t-testing and ANOVA (Dunnett's ST-test) t-test analysis with *p* < 0.01 denoting a very significant difference, *p* < 0.05, a significant difference, and *p* > 0.05 a non-significant difference.

## Results

### Blood routine, physiological and biochemical indices

Compared with the control group, the counts of lymphocytes, monocytes, granulocytes, the hemoglobin concentration and lymphocyte percentage in the blood of Songliao Black pigs in the experimental groups increased (*p* < 0.05). There was no difference in any other index (*p* > 0.05). Compared with the number of leukocytes, lymphocytes, monocytes, granulocytes and their percentages, the above increased indices of group II were higher than those of groups I and III, i.e., 2% dietary *Eucommia* polysaccharide had the greatest effect, compared with 1, or 3% (Table 1).

**Table 1:** Determination results of blood routine indices of Songliao Black pigs.

Detection indicator	Control group	Test group I	Test group II	Test group III
White blood cell count/(10^9^ l^−1^)	16.50 ± 2.12	15.96 ± 2.05	16.83 ± 2.15	15.69 ± 2.50
Lymphocyte count/(10^9^ l^−1^)	8.36 ± 1.48	9.12 ± 2.18*	9.23 ± 2.60*	9.21 ± 1.01*
The number of monocytes/(10^9^ l^−1^)	1.65 ± 0.35	1.82 ± 0.36*	1.98 ± 0.63*	1.89 ± 0.56*
Granulocyte count/(10^9^ l^−1^)	4.61 ± 0.45	6.10 ± 1.00*	6.57 ± 1.44*	6.35 ± 1.36*
Lymphocyte percentage/%	52.30 ± 2.26	60.33 ± 6.99*	61.12 ± 4.76*	60.77 ± 2.87*
Monocyte percentage/%	10.85 ± 3.61	8.09 ± 1.90	9.89 ± 2.22	9.55 ± 2.80
Granulocyte percentage/%	31.59 ± 6.72	36.85 ± 1.34	37.78 ± 2.43	37.48 ± 1.55
Hemoglobin concentration/(g l^−1^)	117.80 ± 1.70	152.44 ± 3.42*	149.12 ± 2.34*	150.35 ± 1.84*
Average hemoglobin content/pg	23.60 ± 0.99	25.56 ± 0.97	26.32 ± 1.02	24.45 ± 0.77
Average hemoglobin concentration/(g l^−1^)	376.50 ± 6.36	389.14 ± 14.62	374.25 ± 12.44	368.33 ± 7.26
Erythrocytes/(10^12^ l^−1^)	4.99 ± 0.14	5.93 ± 1.13	5.21 ± 1.15	5.43 ± 1.29
Average red blood cell volume/fL	62.70 ± 1.70	65.79 ± 2.54	65.65 ± 3.87	64.32 ± 1.45
Hematocrit/%	31.25 ± 0.07	38.93 ± 7.32	35.45 ± 8.65	37.38 ± 3.23
Red blood cell distribution width SD/fL	28.50 ± 0.71	29.86 ± 1.21	27.24 ± 1.11	28.68 ± 0.87
Red blood cell distribution width CV/%	11.30 ± 0.14	11.31 ± 0.29	12.30 ± 0.92	12.12 ± 0.32
Platelet count/(10^9^ l^−1^)	69.00 ± 15.56	82.86 ± 13.75	81.98 ± 16.57	80.45 ± 18.26
Platelet hematocrit/%	0.07 ± 0.02	0.10 ± 0.03	0.09 ± 0.01	0.09 ± 0.04
Average platelet volume/fL	10.60 ± 0.57	10.87 ± 0.44	10.91 ± 0.14	10.45 ± 0.88
Platelet distribution width/fL	16.45 ± 2.90	17.14 ± 2.10	17.98 ± 2.97	18.41 ± 4.23
Large platelet ratio/%	24.80 ± 6.65	28.46 ± 4.59	27.58 ± 4.79	28.32 ± 3.58

Asterisks indicate that the difference between coefficients for the experimental group and control group are statistically significant at *P < 0.05 and **P < 0.01.

Compared with the control group, the concentrations of albumin and total protein in the serum of the experimental groups increased (*p* < 0.01); the contents of urea nitrogen and triglyceride decreased (*p* < 0.05); the contents of globulin and glucose were not different (*p* > 0.05). There was no difference in these biochemical indices among the three groups (*p* > 0.05) (Table 2).

**Table 2:** Determination results of serum biochemical indices of Songliao Black pigs.

Detection indicator	Control group	Test group I	Test group II	Test group III
Total protein/(g l^−1^)	59.86 ± 2.67	74.40 ± 7.10**	76.47 ± 5.24**	77.87 ± 5.15**
Albumin/(g l^−1^)	31.00 ± 3.87	44.56 ± 2.80**	49.44 ± 4.84**	48.18 ± 1.12**
Globulin/(g l^−1^)	28.86 ± 3.89	30.80 ± 4.20	29.78 ± 2.04	32.42 ± 2.44
Urea nitrogen/(mmol l^−1^)	5.43 ± 0.81	3.70 ± 0.80*	3.58 ± 1.08*	4.12 ± 1.22*
Triglyceride/(mg dl^−1^)	48.86 ± 11.54	27.05 ± 4.20*	25.85 ± 5.42*	26.77 ± 2.04*
Glucose/(mmol l^−1^)	3.90 ± 0.10	3.11 ± 1.09	3.54 ± 1.45	3.35 ± 2.26

Asterisks indicate that the difference between coefficients for the experimental group and control group are statistically significant at *P < 0.05 and **P < 0.01.

### Immunoglobulin and cytokine assays

**Figure 1:**
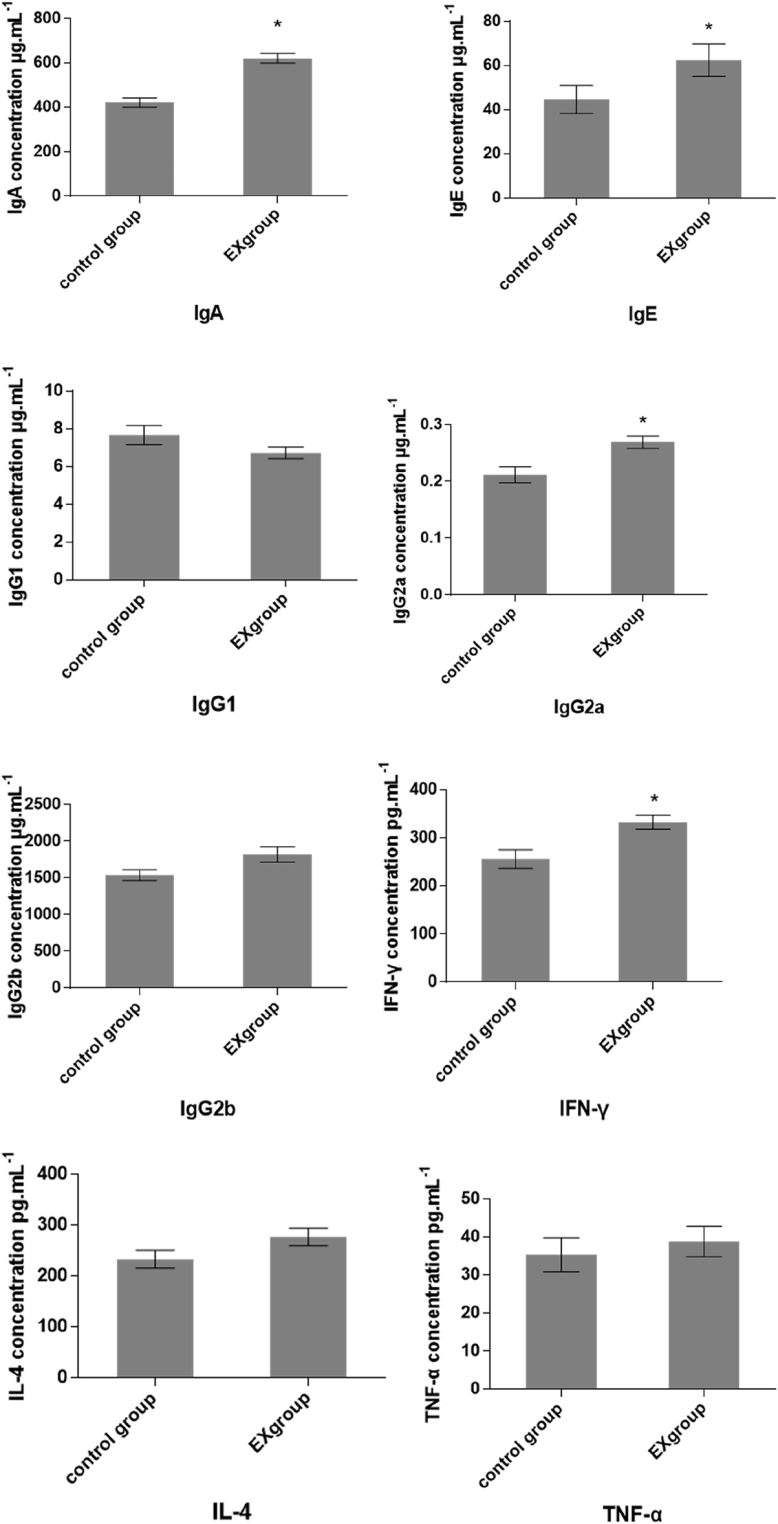
Effect of *Eucommia ulmoides* Oliv. polysaccharide on immunoglobulin and cytokine levels of Songliao Black pigs. Asterisks indicate that the difference between coefficients for the experimental group and control group are statistically significant at *P < 0.05 and **P < 0.01.

Compared with the control group, the serum levels of immunoglobulins IgA, IgE and its subclass IgG2a of pigs in the experimental groups increased (*p* < 0.05), and that of IgG2b increased slightly (*p* > 0.05; Fig. 1). The serum level of IFN-γ in the experimental groups increased (*p* < 0.05), and those of IL-4 and TNF-α increased slightly (*p* > 0.05).

### Growth performance

There was no difference between the final average weight of the experimental groups (*p* > 0.05; Table 3). There was also no difference between the daily weight gain of group I and the control group (*p* > 0.05), whereas group II was higher than control (*p* < 0.05). There was no difference in daily feed intake between the groups (*p* > 0.05). The material to weight ratios of groups I and II were lower than control (*p* < 0.01).

**Table 3:** Effect of *Eucommia ulmoides* Oliv. polysaccharide on growth performance of Songliao Black pigs.

Projects	Control group	1% *Eucommia ulmoides* polysaccharide group	2% *Eucommia ulmoides* polysaccharide group
Initial weight (kg)	83.10 ± 3.67	85.65 ± 3.66	83.15 ± 4.29
End weight (kg)	107.20 ± 3.34	111.40 ± 5.52	109.60 ± 5.37
Daily gain (g)	803.33 ± 54.67	858.33 ± 76.47*	881.67 ± 88.33*
Average daily feed intake (kg)	3.67 ± 0.13	3.24 ± 0.12	3.29 ± 0.06
Material-weight ratio	4.59 ± 0.32	3.81 ± 0.36 **	3.77 ± 0.39**

Asterisks indicate that the difference between coefficients for the experimental group and control group are statistically significant at *P < 0.05 and **P < 0.01.

### Carcass performance

There was no difference in the live weight before slaughter between the groups (*p* > 0.05; Table 4). The slaughter rate and lean meat rate group II were higher than control (*p* < 0.05), whereas the fat rate was lower (*p* < 0.01). Except for carcass weight, there was no difference between groups I and II and control (*p* > 0.05). There were no differences in carcass length, carcass oblique length, skin thickness, eye muscle area, bone rate and skin rate between the groups (*p* > 0.05).

**Table 4:** Effect of *Eucommia ulmoides* Oliv. polysaccharide on carcass performance of Songliao Black pigs.

Projects	Control group	1% *Eucommia ulmoides* polysaccharide group	2% *Eucommia ulmoides* polysaccharide group
Number of samples/head	3	3	3
Live weight before slaughter (kg)	109.33 ± 0.58	115.33 ± 1.53	114.67 ± 2.08
Carcass weight (kg)	76.40 ± 1.65	80.97 ± 0.98*	81.68 ± 1.06**
Slant length of carcass (cm)	74.50 ± 2.29	76.67 ± 1.53	76.83 ± 2.02
Straight length of carcass (cm)	97.33 ± 1.53	98.33 ± 3.06	98.33 ± 1.53
Average back fat thickness (cm)	3.20 ± 0.15	3.10 ± 0.13	3.09 ± 0.13
Thickness of skin (mm)	2.83 ± 0.31	2.81 ± 0.21	2.38 ± 0.16
Eye muscle area (cm^2^)	28.69 ± 0.73	29.83 ± 0.86	31.63 ± 3.84
Slaughter rate (%)	68.62 ± 0.98	70.21 ± 0.61*	71.24 ± 0.42*
Lean meat percentage (%)	57.67 ± 0.96	58.65 ± 0.86*	60.44 ± 1.04*
Fat percentage (%)	19.98 ± 0.37	19.88 ± 0.81	18.53 ± 0.28 **
Bone rate (%)	12.57 ± 0.27	12.41 ± 0.68	11.81 ± 0.93
Skin rate (%)	9.79 ± 0.87	9.06 ± 0.12	9.23 ± 0.41

Asterisks indicate that the difference between coefficients for the experimental group and control group are statistically significant at *P < 0.05 and **P < 0.01.

### Meat quality

Compared with control, the PH_24_ of group II decreased (*p* < 0.05; Table 5). The yellowness (CIELAB b^#^ value) and centrifugal water loss rate of group II were lower than control (*p* < 0.01), whereas the cooked meat rate was higher (*p* < 0.05). The centrifugal water loss of group I was lower than control (*p* < 0.05). The brightness and redness (CIELAB L^#^ and a^#^), meat color score, marbling score, drip loss, water loss rate, shear force and intramuscular fat varied slightly between the groups (*p* > 0.05).

**Table 5:** Effect of *Eucommia ulmoides* Oliv. in feed on meat quality of Song Liao Black Pork.

Projects	Control group	1% *Eucommia ulmoides* polysaccharide group	2% *Eucommia ulmoides* polysaccharide group
pH_24_	5.61 ± 0.02	5.51 ± 0.07	5.49 ± 0.05*
Meat color (24 h)			
L^#^	53.01 ± 1.38	49.10 ± 2.51	47.32 ± 1.11
a^#^	6.73 ± 1.15	6.36 ± 0.66	6.15 ± 0.44
b^#^	7.99 ± 0.50	5.32 ± 0.34 **	5.99 ± 0.55**
Meat color score	3.17 ± 1.04	2.33 ± 0.29	2.67 ± 0.29
Marble score	2.50 ± 0.50	3.17 ± 0.76	2.83 ± 0.58
Drip loss (%)	3.51 ± 0.58	2.54 ± 0.78	2.20 ± 0.88
Centrifugal water loss rate (%)	30.33 ± 1.47	22.58 ± 4.37*	21.69 ± 2.53**
Water loss rate (%)	35.23 ± 4.37	30.91 ± 5.44	27.58 ± 4.07
Shear force (n)	37.59 ± 2.78	36.03 ± 1.26	35.92 ± 1.53
Cooked meat rate (%)	60.59 ± 1.16	61.93 ± 1.41	63.59 ± 0.60*
Intramuscular fat (%)	2.72 ± 0.49	3.42 ± 0.19	3.40 ± 0.18

L^#^ stands for brightness, a^#^ for redness and b^#^ for yellowness. Asterisks indicate that the difference between coefficients for the experimental group and control group are statistically significant at *P < 0.05 and **P < 0.01.

### RNA quality and RNA sequencing quality

The RNA quality test showed that the total amount of RNA in 5-1/T1, 6-1/T2, 7-3/T3 and 8-3/C1, 9-4/C2 and 10-1/C3 of the experimental groups, the RNA integrity index (≥ 7.7), and the rRNA ratio (≥ 1.0) met the requirements for database establishment. The numbers of reads from the three control high-throughput sequencing samples were 45,911,760, 47,517,310 and 48,766,628, respectively, and the effective rates were 97.87, 96.68 and 97.76% respectively. The numbers of reads from the three experimental group samples were 48,449,042, 46,867,530 and 45,715,882, and the effective rates were 97.73, 97.85 and 97.50%, respectively. The data comparison rate and effective rate were above 95% (Table 6) and the ratios of base quality (Q30) of the sequencing were all over 93%, indicating good sequencing quality that met the quality control requirements.

**Table 6:** Significant differential expression of genes in 2% *Eucommia ulmoides* polysaccharide group.

Gene name	Difference multiple (FC)	Up/down
ENSSSCG00000004602 (TEX9)	− 1.293353565	Down
ENSSSCG00000006359 (ADAMTS4)	2.63164307	Up
ENSSSCG00000010219 (ARID5B)	1.601829628	Up
ENSSSCG00000010370	1.633814652	Up
ENSSSCG00000012880 (CPT1A)	− 2.178109843	Down
ENSSSCG00000013599 (ANGPTL4)	− 1.384130813	Down
ENSSSCG00000013784 (DNAJB1)	1.781511378	Up
ENSSSCG00000014834 (UCP3)	− 2.465414635	Down
ENSSSCG00000016605 (LMOD2)	1.610635534	Up
ENSSSCG00000016646 (IFRD1)	1.385607506	Up
ENSSSCG00000016690 (CREB5)	1.468009525	Up
ENSSSCG00000017983 (PER1)	2.150248558	Up
ENSSSCG00000022256 (C10orf10)	1.320485504	Up
ENSSSCG00000022300 (MAT2A)	1.193117061	Up
ENSSSCG00000022925	1.601496151	Up
ENSSSCG00000023085 (STAC)	2.632826616	Up
ENSSSCG00000025460 (SPSB2)	− 2.164376564	Down
ENSSSSCG00000025698 (SERPINE1)	3.067596632	Up
ENSSSCG00000029226	1.092500192	Up
ENSSSCG00000029571 (AVIL)	1.69481585	Up
ENSSSCG00000029944 (FASN)	2.859912751	Up
ENSSSCG00000031450	− 2.30582212	Down
ENSSSCG00000031912	1.64860759	Up
ENSSSCG00000032527 (FOSL2)	1.384061425	Up
ENSSSCG00000032810 (GZMM)	1.6163484	Up
ENSSSCG00000033822 (THRSP)	2.800041411	Up
ENSSSCG00000034102 (DGAT2)	1.425353251	Up
ENSSSCG00000034328 (SP7)	5.085327819	Up
ENSSSCG00000036742 (KLF15)	1.601574701	Up
ENSSSCG00000038598 (ADRB2)	1.546495169	Up
ENSSSSCG00000039468 (SERPINH1)	1.301188801	Up
ENSSSCG00000040636 (KRT80)	2.410615016	Up

### Differential gene analysis

**Figure 2:**
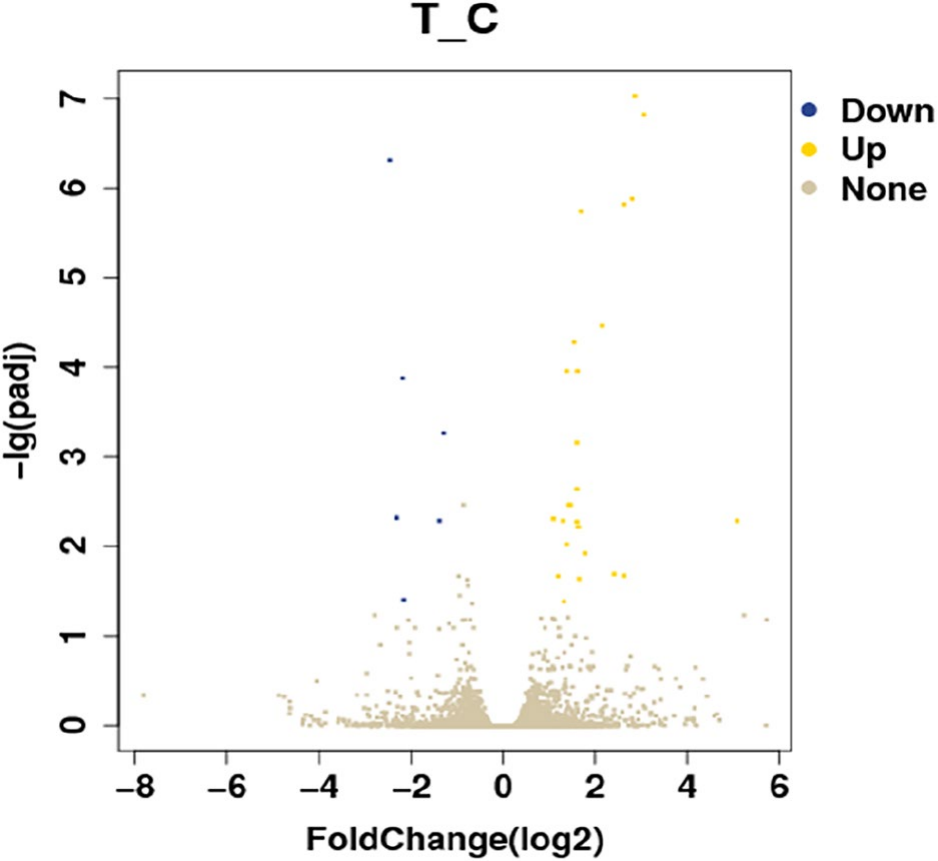
Differential gene volcano map. The abscissa is the logarithmic value of differential gene expression multiple in the comparison group, and the ordinate is the negative logarithmic value of the statistical significance of expression change; Each dot represents a gene, and the genes that are significantly up-regulated (yellow), significantly down-regulated (blue) and have no significant difference (gray) are marked with different colors.

**Figure 3:**
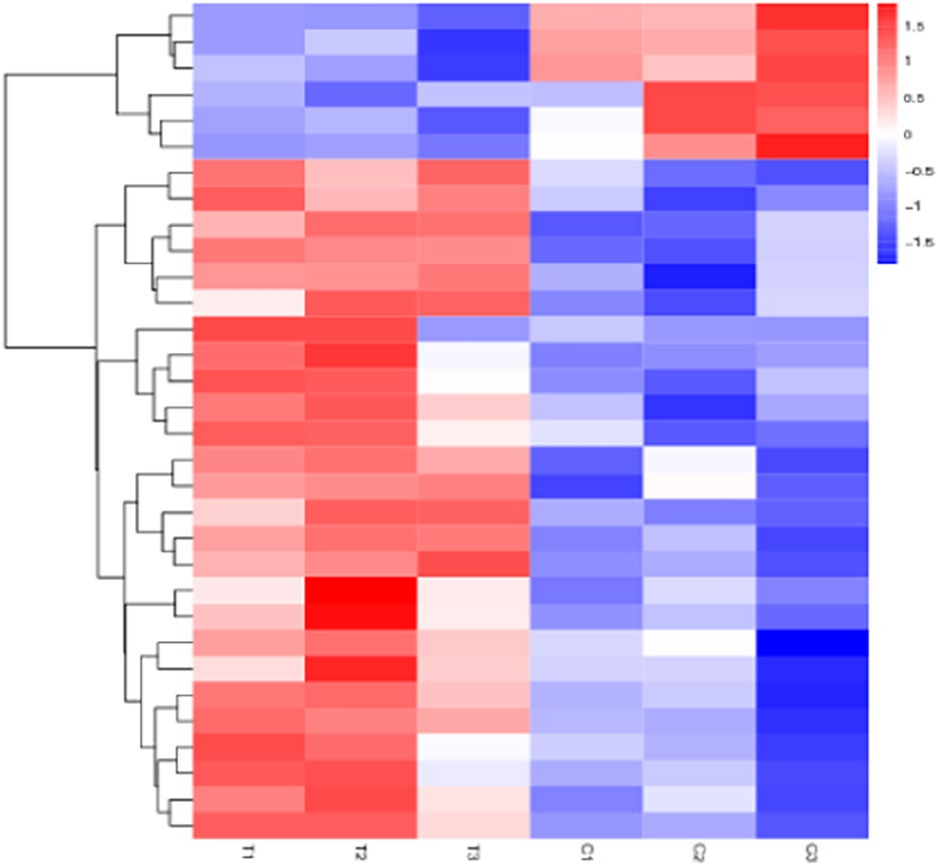
Differential gene clustering map. Different columns represent different samples and different rows represent different genes. Color indicates the expression level of genes.

**Figure 4:**
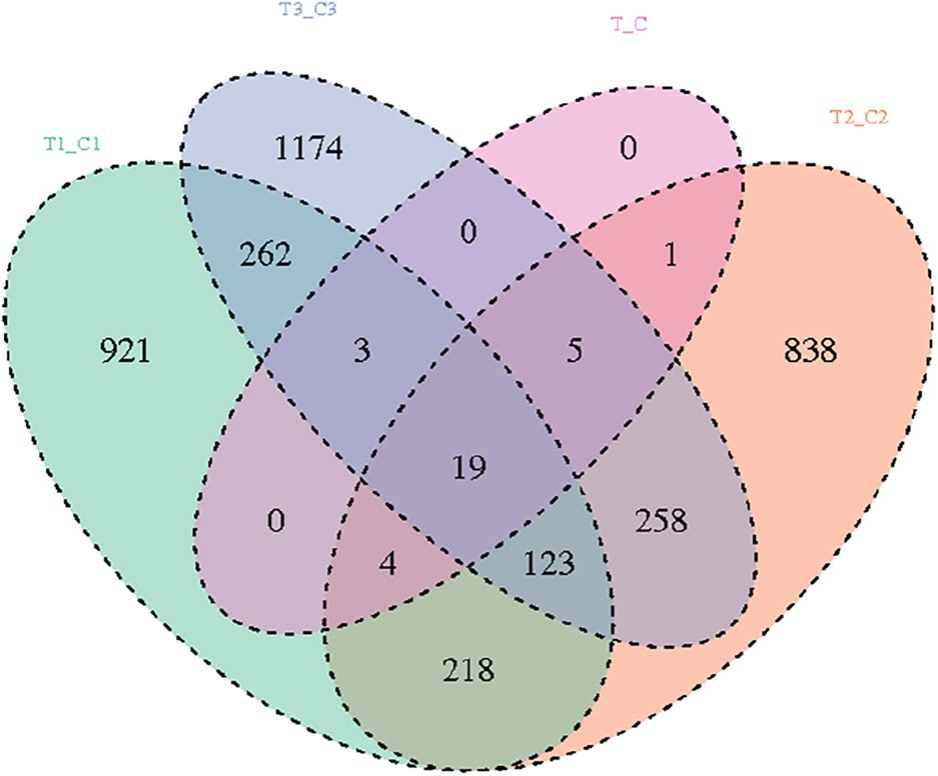
Wayne diagram of differential genes between groups. The sum of numbers in each circle represents the total number of differential genes in the comparison group, the overlapping part of the circle represents the common differential genes among the combinations, and only the part in one circle represents the specific expression of differential genes in the comparison group.

Screening the DEGs with FC ≥ 1 and q < 0.05 between the experimental and control groups identified 32 DEGs (Fig. 2). A hierarchical cluster analysis of the DEGs and treatment conditions was carried out (Fig. 3), finding that 26 genes were up-regulated and six were down-regulated. A total of 19 genes was differently expressed among the four groups, of which 18 were up-regulated (Fig. 4). There were eight DEGs with difference multiples of > 2, i.e., ADAMTS4, PER1, STAC, SERPINE1, FASN, THRSP, SP7 and KRT80, indicating that *Eucommia* polysaccharide had a significant influence on fat deposition genes.

### Significant enrichment analysis of GO functions and KEGG pathways of DEGs

**Figure 5:**
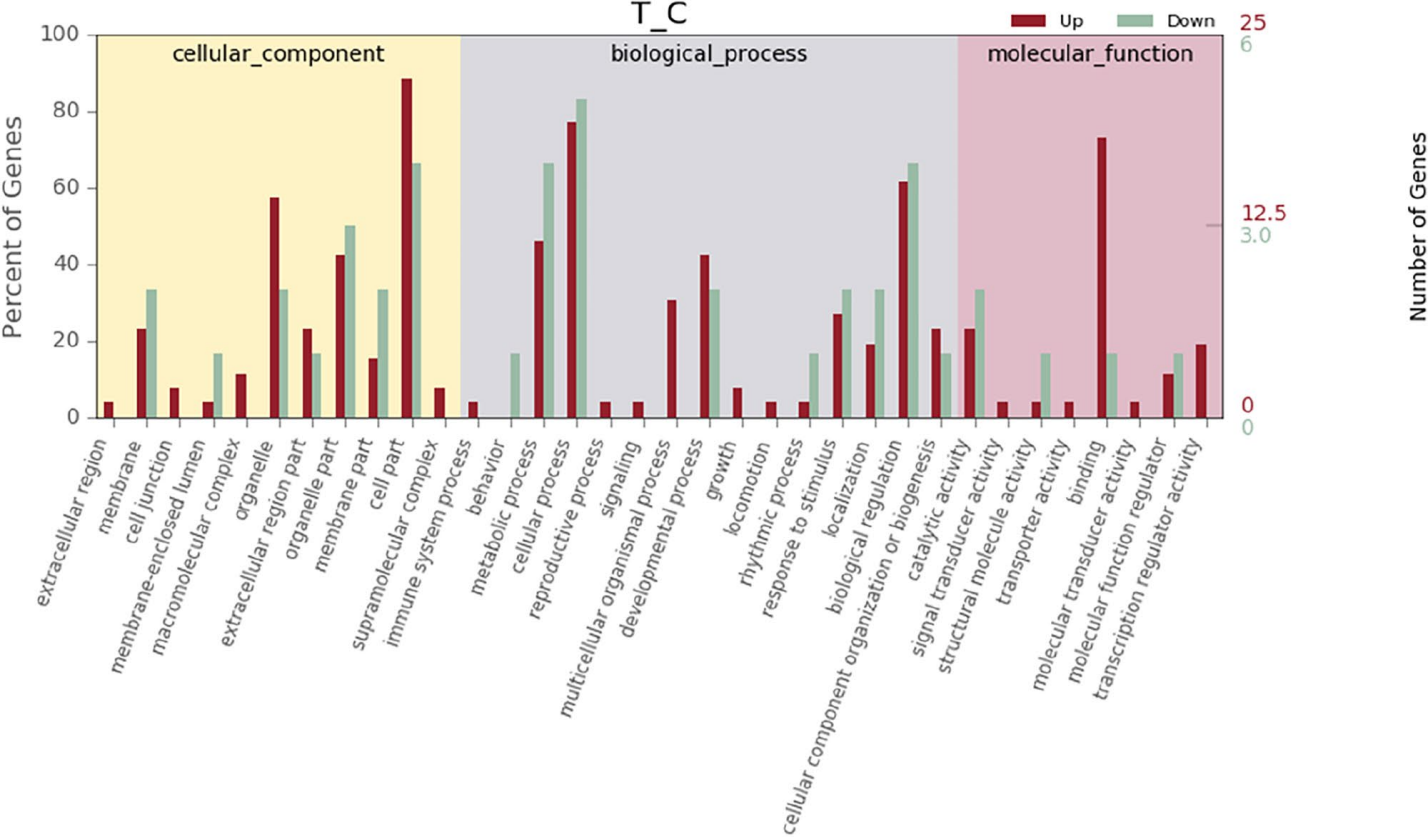
GO statistical histogram of differently expressed genes. The abscissa is GO item, the left ordinate is the percentage of gene number, and the right ordinate is the gene number.

**Figure 6:**
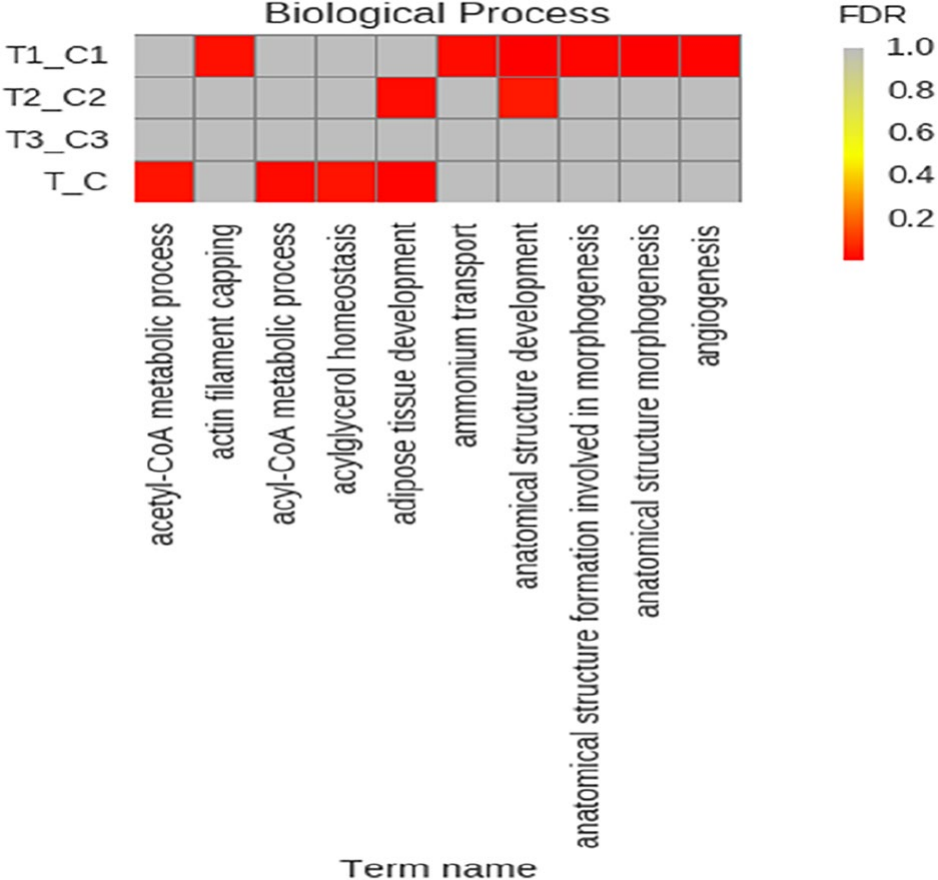
Distribution diagram of Q value of enriched GO items. The ordinate shows the names of different comparison groups, the abscissa shows the entries of GO, and different colors represent different degrees of enrichment.

**Figure 7:**
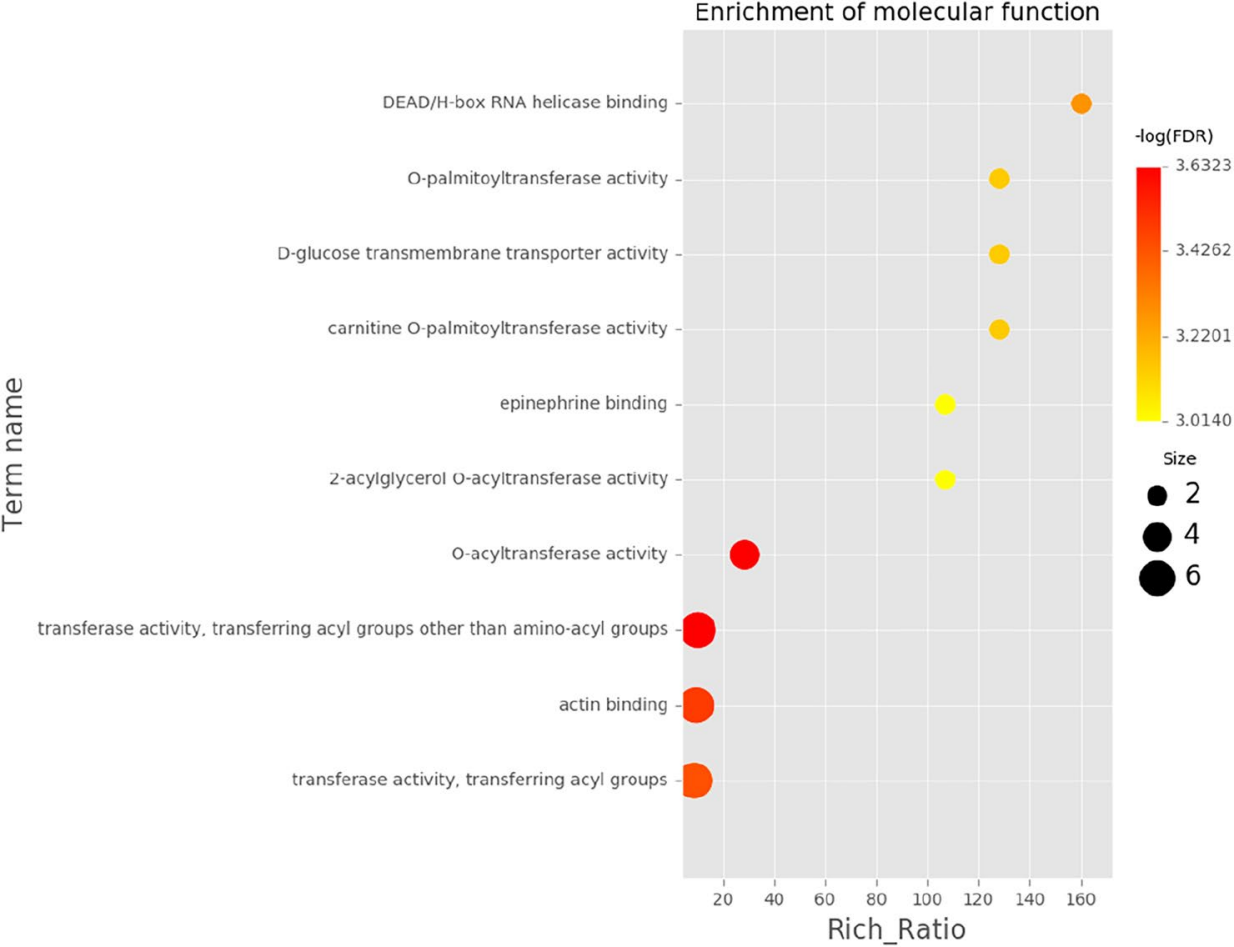
Enrichment analysis of differently expressed GO items**.** The abscissa represents enrichment ratio, and the ordinate represents different GO items. The color of the dot indicates the enrichment degree of the GO item, and the size indicates the number of genes enriched to the GO item.

**Figure 8:**
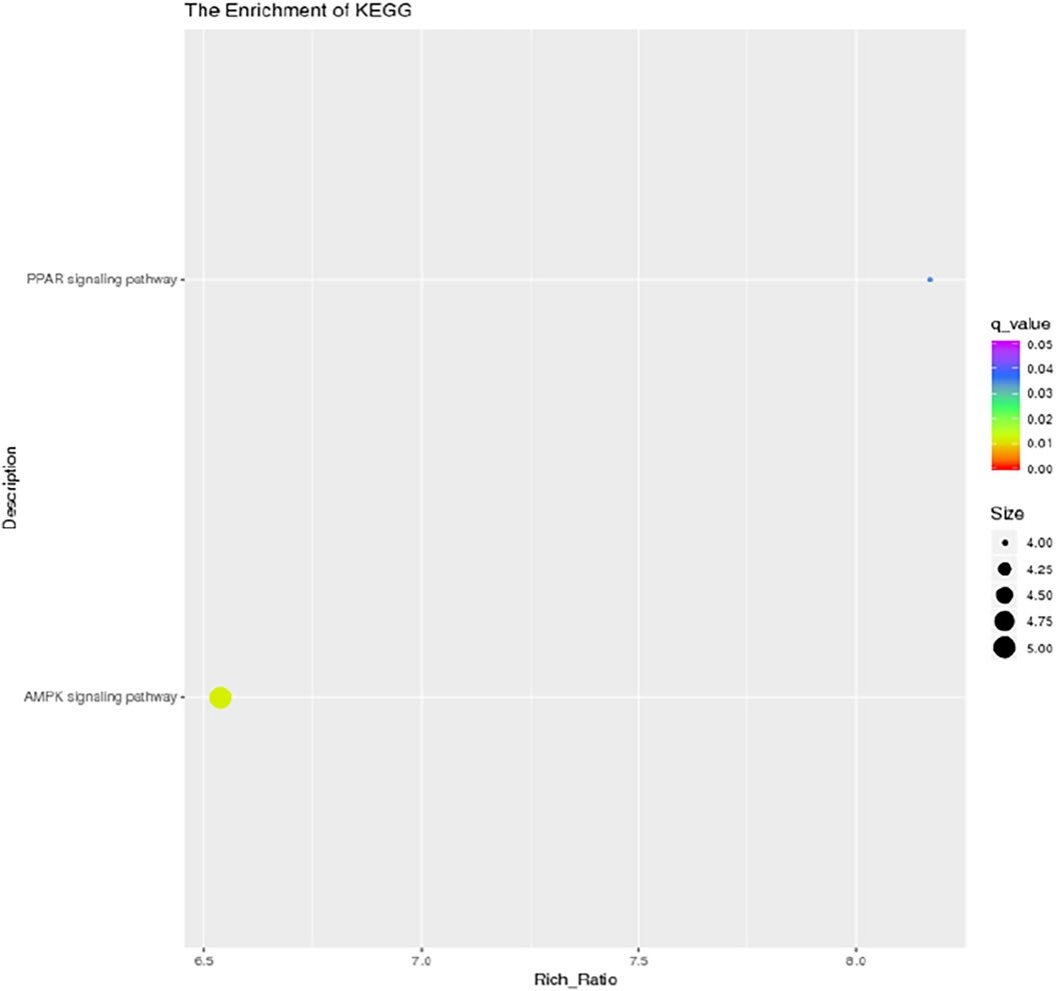
KEGG pathway analysis of differential genes. The abscissa indicates the enrichment factor, the ordinate indicates the KEGG pathway name, the color of the dot represents the enrichment degree of the KEGG entry, and the size represents the number of differential genes in the pathway.

**Figure 9:**
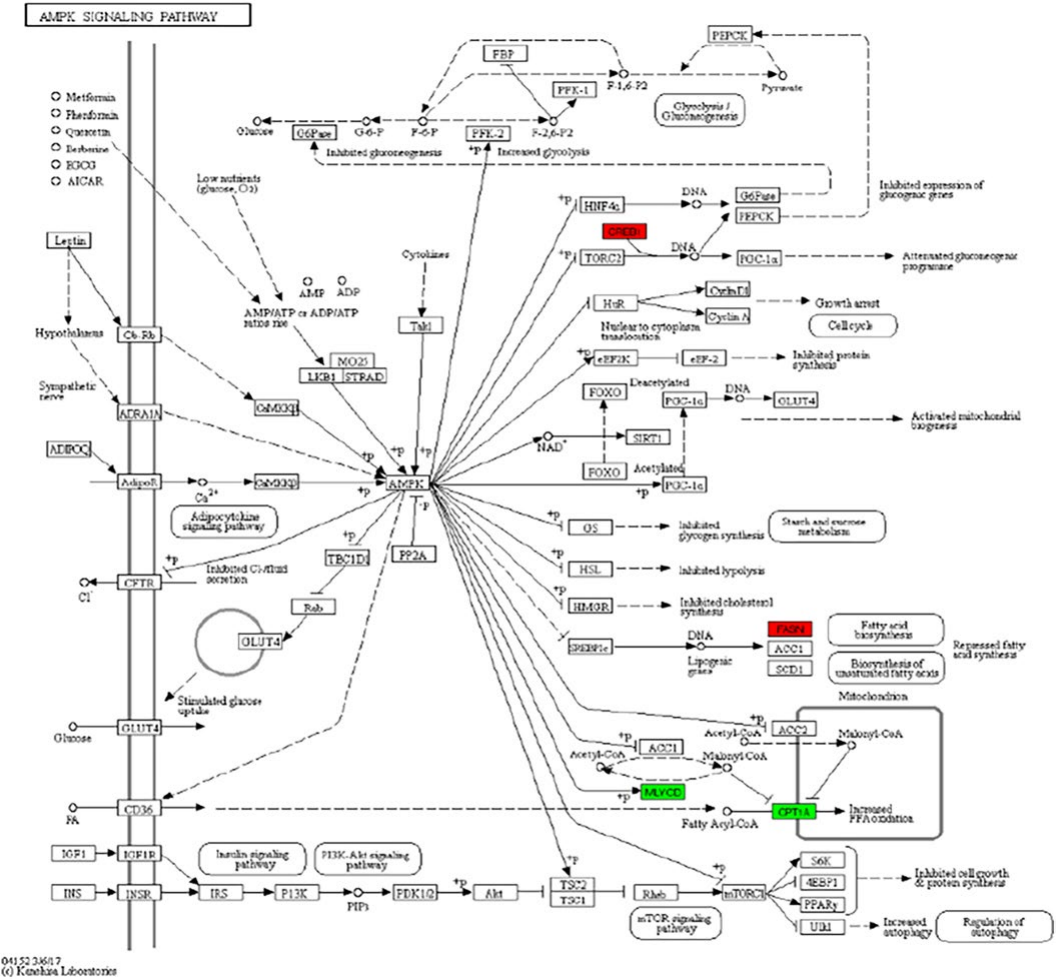
AMPK signal path. Red is up-regulated gene and green is down-regulated gene.

**Figure 10:**
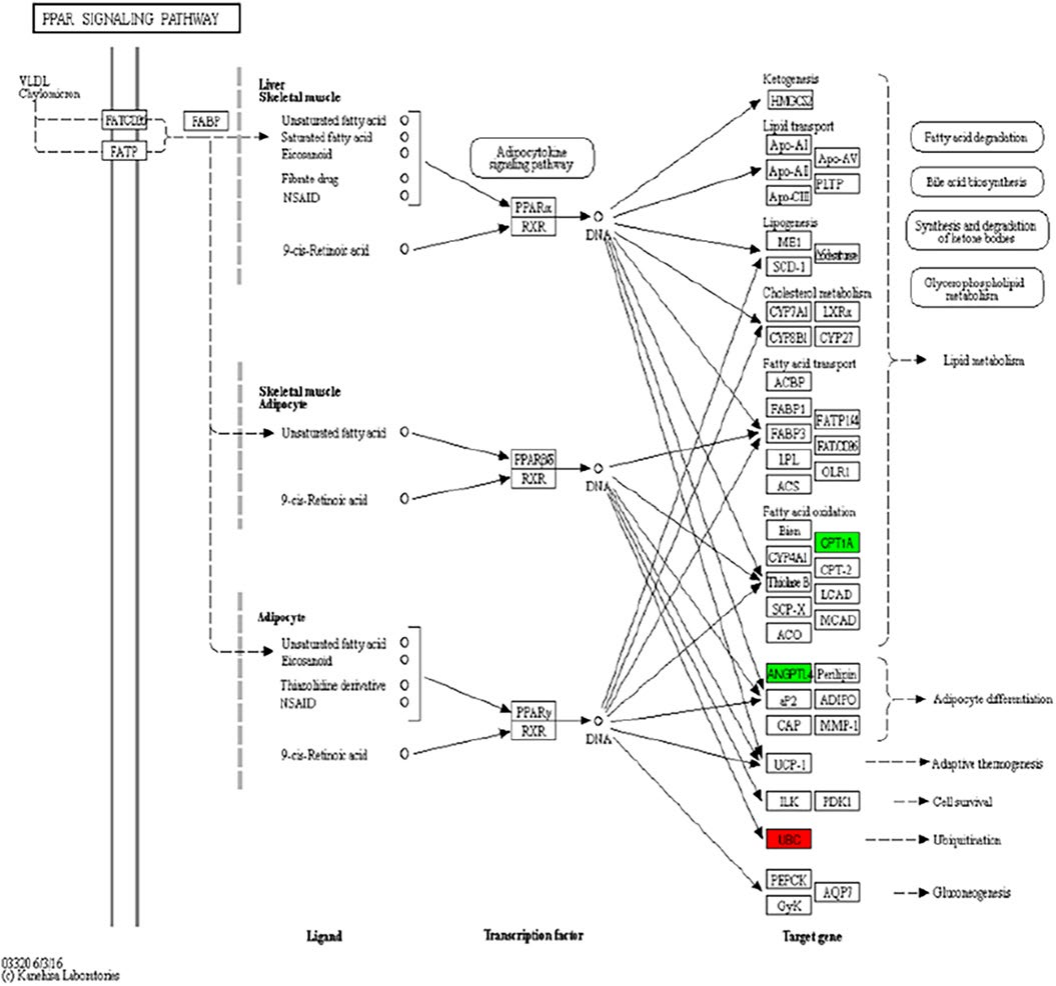
PPAR signal path. Red is up-regulated gene and green is down-regulated gene.

GO function annotation (Fig. 5) and enrichment analysis (Figs. 6, 7) were performed on the above 32 DEGs, of which 34 were significantly enriched in transferase activity, actin binding, among which acetyl coenzyme A, acyl coenzyme A metabolism, adipose tissue development and acyl glycerol homeostasis were significantly different. KEGG analysis (Fig. 8) showed that differential genes were significantly enriched in the AMPK (Fig. 9) and PPAR (Fig. 10) signaling pathways. These results indicate that *Eucommia* polysaccharide had a significant effect on intramuscular fat deposition.

### Fluorescence quantitative PCR to validate gene expression analysis

**Figure 11:**
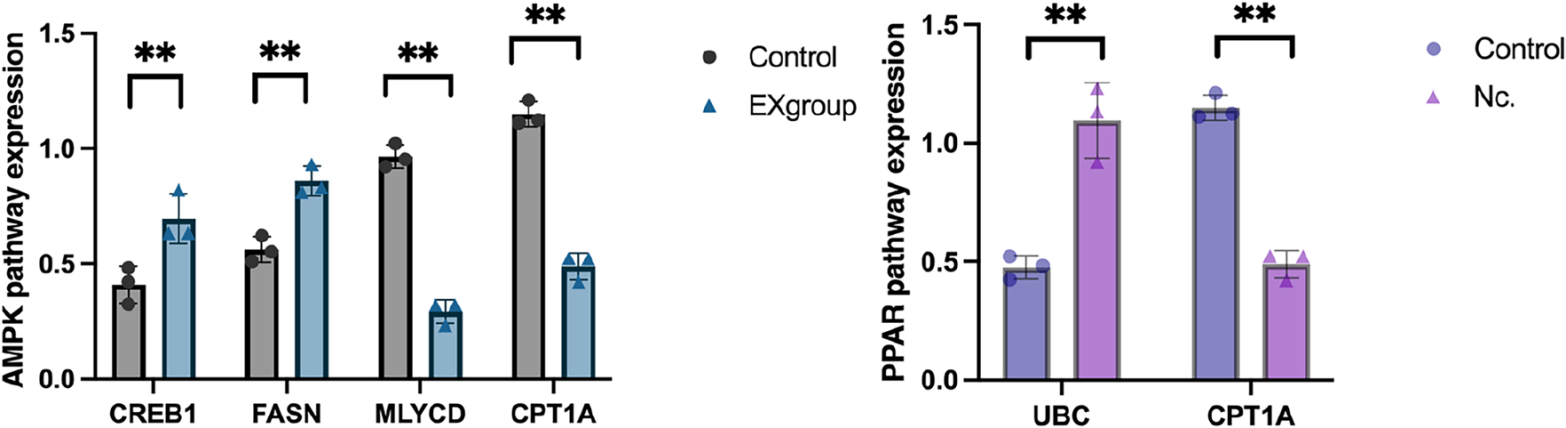
RT-qPCR validation of RNA-seq sequencing results. (**A**) AMPK signal path; (**B**) PPAR signal path. *P < 0.05, **P < 0.01 vs. control group.

The RNA expression changes of five genes in the AMPK signalling pathway and PPAR signalling pathway were validated and analysed using real-time fluorescence quantitative PCR. The results showed that the RT-qPCR results were consistent with the expected results from sequencing. In the AMPK signalling pathway, CREB1 and FASN were significantly up-regulated, and MLYCD and CPT1A were significantly down-regulated; in the PPAR signalling pathway, UBC was significantly up-regulated and CPT1A was significantly down-regulated (Figs. 9, 10, 11).

## Discussion

Changes in levels of blood components reflect changes in metabolic capacity and physiological activity. At the initial stage of infectious disease, the neutrophil count increases greatly, which is known as "the first line of life defense" and reflects the health of the organism. An increase in hemoglobin content is beneficial for the full utilization of oxygen in a hypoxic environment. Dietary *Eucommia ulmoides* Oliv. polysaccharide (*Eucommia*) significantly increased the blood counts of lymphocytes, monocytes, granulocytes, and the hemoglobin concentration in Songliao Black pigs. Although there was no significant difference in other indices, they all showed an upward trend, indicating that *Eucommia* was beneficial to the health status of the pigs. Taken together, the above blood indices, indicate that experimental group II (2% *Eucommia*) received greater benefits than groups I and III (1 and 3%).

Serum biochemical indicators reflect the metabolic state and health status of animals, as well as changes in protein and lipid metabolism. Generally, increased serum total protein and albumin content indicates enhanced liver protein synthesis, and the triglyceride metabolism level is related to cardiovascular and cerebrovascular diseases. The level of urea nitrogen reflects the amino acid balance. Dietary *Eucommia* extract significantly reduced blood urea nitrogen and triacylglycerol concentration in Duchangda ternary hybrid pigs^14^ and increased total serum protein in Duchangsanyuan piglets^15^. This study found that dietary *Eucommia* significantly increased serum total protein and albumin in Songliao Black pigs, and significantly decreased serum urea nitrogen and triglyceride, indicating that *Eucommia* increased liver protein synthesis and enhanced overall health.

Blood immune mediators are markers for immune system function and general health. Dietary *Eucommia* leaf extract and its fermentation products increased serum IgM in weaned piglets, and slightly increased the IgG concentration^8^. Dietary *Eucommia* leaves increased serum IgA and IgG in piglets^16^. This study showed that 2% dietary *Eucommia* polysaccharide increased serum IgA, IgE and its subclass IgG2a in Songliao Black pigs, indicating enhanced immune function and overall health.

Cytokines regulate immune function, for examples, interferon is a multifunctional active glycoprotein, which inhibits invasion, shelling, replication and release of some viruses; Interleukin-4 (IL-4) can up-regulate the growth of B cells and immunoglobulin secretion, thereby strengthening the humoral immune response, but it can also inhibit the differentiation of Th1 cells and enhance the differentiation of Th2 cells, an immune-suppressive effect. Increased TNF-α stimulates macrophage activation, which enhances their function of killing tumor cells. This study showed that 2% dietary *Eucommia* polysaccharide increased the levels of cytokines IFN-γ, IL-4 and TNF-α, indicating improved general immune function and specific immune function against viruses and tumors.

*Eucommia* bark and leaves can improve animal productivity, promote lipid metabolism and protein synthesis, and improve meat quality and flavor. Dietary *Eucommia* increased the intramuscular fat content of Ningxiang castrated sows^17^, and stabilized the pH, increased the tenderness and improved the taste of meat from Du × Long × Big three-way crossbred pigs^18^. This study showed that 2% dietary *Eucommia* increased the average daily weight gain, slaughter rate, lean meat rate and cooked meat rate, and decreased pH_24_, feed-to-weight ratio, fat rate, yellowness (b^#^) and centrifugal water loss, indicating that dietary *Eucommia* improved the meat quality of Songliao Black pigs.

The effect of dietary *Eucommia* polysaccharide on the genes related to intramuscular fat deposition in Songliao Black pigs was investigated. Analysis and comparison of differently expressed genes (DEGs) identified eight genes with a > twofold change in expression, of which, ADAMTS4, PER1, FASN and THRSP are involved in intramuscular fat deposition. After knockout of the ADAMTS4 gene, the mRNA expression levels of PPAR, dgat2, COL3A1, adamts2, ADAMTS4 and aggrecan genes related to adipocyte differentiation were down regulated^19^. PER1 regulates changes in mitochondrial crista state, mitochondrial function and lipid absorption^20^. RNA-seq and GO analysis of DEGs related to intramuscular fat deposition in the longissimus dorsi muscle of pigs showed that the fat metabolism mechanism mediated by FASN was closely related to the intramuscular fat ratio, and the expression of FASN increased the intramuscular fat ratio, thus enhancing meat quality^21^. High expression of THRSP may provide sufficient glucose raw material for fat synthesis, by regulating GLUT4 transcription, and accelerate fat production by regulating FASN and ACC transcription^22^. In this study, the expression levels of ADAMTS4, PER1, FASN and THRSP were markedly increased, indicating that dietary *Eucommia* polysaccharide improves intramuscular fat deposition in Songliao Black pigs.

Protein interaction analysis showed that the differently expressed proteins were of two types, one related to the immune system and the other related to fat deposition. This is also illustrated by the ability of dulcimer powder to enhance the immune function and improve the meat quality traits of Songliao black pigs in this study. Osteoblasts isolated from mice with a Fosl 2 gene deletion were cultured in vitro, resulting in increased expression of the lipogenesis signal genes C/EBP-α, C/EBP-β and PPAR-γ^23^. The expression level of PPAR-γ, C/EBPα and AP2 decreased markedly and the triglyceride concentration decreased markedly after the expression of KLF15 decreased; lipid droplets in adipose tissue decreased in size and quantity^24^. This study found that dietary *Eucommia* significantly increased expression of the FOSL2 and KLF15 proteins, consistent with the above reports.

The AMPK signaling pathway mainly regulates the cellular balance of energy and metabolism, and participates in the regulation of fat synthesis by influencing the synthesis of the adipogenic mediators PPAR-γ, C/EBP-α, SREBP1c and FAS^25^. PPAR-α is active in skeletal muscle and adipose tissue, participates in regulating fat metabolism and is associated with intramuscular fat deposition^26^. PPAR-γ mainly regulates the differentiation of adipocytes and maintains the function of mature adipocytes^27^. GO function and KEGG pathway analysis of the identified DEGs found that PPAR-γ, C/EBP-α, SREBP1c and FAS were mainly involved in transferase activity and actin binding, and are significantly enriched in the AMPK and PPAR pathways related to fat deposition, in which FASN, a gene related to fat synthesis, was significantly up-regulated.

## Conclusion

Dietary *Eucommia ulmoides* polysaccharide (2% w/w in pig feed) increased numbers of immune cells, immunoglobulin and cytokine levels, and improved growth performance, carcass performance and meat quality traits in Songliao Black pigs. Dietary *Eucommia* also up-regulated the expression of genes involved in fat deposition, increased fat deposition, enhanced immune function and improved meat quality.

### Supplementary Information

Supplementary Information.

## Data Availability

The datasets generated or analysed during the current study are available in the NCBI (National Center for Biotechnology Information) repository, ACCESSION NUMBER TO DATASETS:ID PRJNA1033230.
