# Supplementary material for: Effect of dietary Eucommia ulmoides oliver polysaccharide on immune function and meat quality of Songliao Black Pigs
Source: Sci Rep. 2024 Jun 17;14:13901. doi: 10.1038/s41598-024-64257-4 (PMC11183226; doi:10.1038/s41598-024-64257-4)
Supplement: Supplementary file 1 — Supplementary Information. [file 41598_2024_64257_MOESM1_ESM.pdf]

Industry standard information service platform
hom Standard query Standard announcement query Monthly report help center
Industry standard record system login entrance

ICS 67.050
X 04

NY

Agricultural Industry standards of the People's Republic of China

NY/T 821—2004

Technical specification for the determination of muscle quality in pigs

Technical regulation for determination of pork quality

2004-08-25 2004-09-01 Implementation

The Ministry of Agriculture of the People's Republic of China

preface

This standard is proposed and centralized by the Ministry of Agriculture of the People's Republic of China.

Drafting units of this standard: Quality Supervision, Inspection and Testing Center of Ministry of Agriculture (Wuhan), Quality Supervision, Inspection and Testing Center of Ministry of Agriculture (Guangzhou).

The main drafters of this standard are: Ni Debin, Xiong Yuanzhu, Deng Changyan, Liu Wanghong, Hu Junyong, Lei Minggang, Liu Xiaohong, Qian Huiyue.

Technical specification for the determination of muscle quality in pigs

1 range

This standard specifies the indicators, methods and conditions for the determination of pig muscle quality.

This standard is applicable to the determination of pig muscle quality.

2. Normative references to the reference documents

The terms in the following documents become the terms of the standard by reference to this standard. All subsequent amendments (excluding errata) or revisions do not apply to this standard. However, the parties agreed under this standard are encouraged to study the availability of the latest version of these documents. The latest version of the undated references applies to this standard.

NY / T825-2004 Specification for determination of carcass traits in lean pigs

3 Terms and definitions

The following terms and definitions are applicable to this standard.

3.1

Meat-color meat color

The bright color of the muscle cross-section.

3.2

Muscle pH muscle pH

The pH of the muscle in a certain time after slaughter, or pH.

3.3

Hydraulic water holding capaci ty, WHC.

The ability of isolated muscles to maintain their internal water content for a certain period of time under certain conditions. According to the different conditions, it is expressed as water potential, available water and dripping loss.

3.3.1

Hydrogenic potential, water holding potential

The muscle'its ability to retain water for a certain period of time.

3.3.2

Can be squeezed out of the water of expressible moisture

Muscle under a certain external force, in the amount of water within the specified time.

3.3.3

Dripping loss of drip loss

The loss of water within the muscle within a specified time without external force.

3.4

Intramuscular fat, intramuscular fat, IMF.

Fat content in the muscle tissue.

3.5

Marbled lines of marbling

The distribution of fat and connective tissue is seen in muscle cross section.

3.6

Muscle quality of meat quality

In the process of conversion from muscle to edible meat, the original various physicochemical characteristics of muscle and the quality characteristics related to consumption and circulation, such as meat color, hydraulic force, pH, flavor, etc., referred to as meat quality.

3.7

PSE pale soft and exudative

Within a certain period of time, the muscle appeared gray (pale), soft (soft) and cut juice extravasation (exudative).

3.8

DFD dark firm and dry

For a certain period of time, the muscles are dark (dark), tight and hard (thin) and dry (dry).

3.9

Acid meat, acid meat

Within 45min after slaughter, the muscle pH_1 was maintained above 6. 1, but then the muscle pH decreased rapidly and the muscle pH_{24} decreased to 5.5 The following phenomenon.

4 Pre-slaughter treatment and slaughter conditions

Press NY / T 825- -2004.

5 for sampling

5.1 Sampling time

Samples should be taken within 30 min after the pigs stopped breathing.

5.2 Sampling site

5.2.1 Take 20 cm to 30 cm from the back of the left half of the left half of the carcass.

5.2.2 Mark on the third to fourth thoracic vertebra from the bottom, and indicate the slaughter time, sampling time, sample number, sample weight and sampling person on the label.

5.2.3 Place in a covered white square porcelain plate for testing.

6 Assay method

6.1 Meat color

6.1.1 Determination method

6.1.1.1The colorimetric plate scoring method

A) Determination time: within 1h~2h after the pigs stopped breathing:

Bb) measured site: the longest dorsal muscle at the thoracolumbar junction:

In c) The meat sample is divided into two, flat in the white porcelain plate, and the meat color rating in natural light, using 6 points color plate score: 1 PSE meat (slightly light red white to white): 2 light PSE meat (light gray red): 3 normal meat color (bright red): 4 normal meat color (dark red): 5, light DFD meat (light purple red): 6 DFD meat (dark purple red):

D) It is appropriate to add 0.5 points between the two integers to record the score value.

6.1.1.2Optical assay

A) Measurement time and site vs6. 1. 1. 1identical:

B) Determination by color differential meter, which shall be equipped with D65 light source with wavelength of 400nm ~700 nm, if measured by using other types of instruments,

Methods and conditions shall be described:

C) Determine the meat sample according to the operation requirements of the instrument, and record the measurement results:

D) Two parallel samples for each meat sample and three points for each parallel sample. The relative deviation between the results of the two parallel samples shall be less than 5%, otherwise it shall be redone immediately.

6.1.2 Description of the measurement results

6.1.2.1The results of the color plate scoring method are expressed as in formula (1):

$$\text{Meat color score} = \text{visual assessment score value} \quad (1)$$

6.1.2.2The results of optical measurement are expressed as in equation (2):

$$\text{Color value} = (2Ri / 3 + 2 Rj / 3) / 2 \quad (2)$$

In formula:

> Ri and Rj —— are the sum of the three readings of each meat sample:

3 —— Number of determinations per meat sample:

2- -Number of meat slices.

6.1.2.3The results were retained to two decimal places.

6.2 Muscle pH

6.2.1 Determination method

6.2.1.1Measurement time: measured within 45min after respiration, recorded as pH_1; 24h after respiration, recorded as pH_{24}.

6.2.1.2Determination site: the longest dorsal muscle from the penultimate first to the second thoracic vertebral segment.

6.2.1.3Remove the peripheral muscle membrane of the meat sample, cut it into small pieces and place it in a clean meat grinder, twisted it into minced meat, and put it into two beakers for testing.

6.2.1.4Using pH meter measurement, pH meter shall be equipped with composite electrode, with precision requirement of 0.01pH: direct plug pH meter can also be used for direct measurement.

6.2.1.5 According to the operation requirements of the instrument, first corrected with pH 4.00 and pH 7.00 standard solution, and then measured with meat samples, separately

Out of pH_1 and pH_{24}, and the determination results were recorded.

6.2.1.6Two parallels were determined for each meat sample, and each parallel sample was determined twice. The relative deviation between the results of two parallel samples shall be less than 5%, otherwise it shall be retested immediately.

6.2.2 Description of the determination results

The test results are expressed as in equation (3):

$$pH = (pHi/2+ pHj/2)2 \quad (3)$$

In formula:

2pHi and pHj —— are the sum of the two measured values of each meat sample, respectively:

2—Meat sample fraction and the number of measurements per fraction of meat sample, respectively.

Asresults are expressed as means and retained to two decimal places.

6.3, the hydraulic force

6.3.1 Determination method

6.3.1.1Dripping loss method

A) Determination time: within 1h~2h after the pigs stopped breathing:

Bb) Determination site: dorsal longest muscle from the 3rd to 4th thoracic vertebra:

C) Sample preparation: the peripheral muscle membrane of meat samples was removed, and the natural muscle fibers were modified into 4cm 4cm 4cm 4 cm meat strips:

D) weigh the weight of each meat strip with a balance and record the number:

E) Hang the end of the meat strip with the hook, put it in the numbered food bag, and expose 1 / 2 of the hook outside the food bag. To fill the food bag, suspend the meat strip in the center of the food bag, and tighten the mouth with the hook with cotton thread, hang it on the rack, and keep it in a 2℃ ~4℃ refrigerator for 48 h:

F) Remove the rack, open the food bag, remove the meat strips, drain the surface moisture with filter paper: then weigh the weight after hanging each strip, and

The number of records.

6.3.1.2pressure application

A) Determination time and measurement site are the same6.3.1.1:

B) Sample preparation: cut 2 pieces of meat about 1cm thick, and take 1 meat sample in the middle of 2 meat pieces with 02.523cm sampler:

Cc) weigh the weight with a balance and record it:

D) Place the meat sample between 2 layers of gauze, pad 18 layers of filter paper and a hard plastic plate on the top and bottom, on the platform of the pressure gauge, pressure to 35 kg, and maintain the removal pressure at 5min:

E) After removing the pressed meat sample, remove the hard plastic plate, filter paper and gauze, weigh the meat sample after pressure, and record it:

F) Two parallel samples for each meat sample, and the relative deviation between the two parallel samples shall be less than 5%, otherwise it shall be reworked immediately.

6.3.2 Description of the measurement results

6.3.2.1The results of drip loss method is as described in equation (4):

$$\text{Dripping loss (\%)} = [(W_1 - W_2) / W_1] 100 \quad (4)$$

In formula:

W_1 —— meat sample hanging front weight, unit in grams (g):

W_2 —— meat sample after hanging weight, unit in grams (g).

6.3.2.2The results of the pressure method are expressed as formula (5), (6), (7) and (8):

Hydraulic (%) = [(water content of meat sample-water loss of meat sample) / water content of meat sample] 100 ...... (5)

Water content of meat sample (g) = preweight (g) Water (%) of the meat sample (see 6.5) (6)

Water loss (g) = weight (g) -weight (g) ...... (7)

Water loss rate (%) = [(weight before-weight after) / weight before] 100 ...... (8)

6.3.2.3Asresults are expressed as means and retained to two decimal places.

6.4 Intramuscular fat

6.4.1 Determination method

6.4.1.1Marble grain scoring method

A) Determination time: within 24 h after the pigs stopped breathing:

B) Determination location is the same6.1.1.1:

C) Sample treatment: store the meat color score sample in 0℃ ~4℃ refrigerator for 24h, divide it into two and place it in a white porcelain plate:

D) According to the 10-point marbling scoring chart, the scoring is visually measured under the natural light conditions, and then the scoring results are recorded. 0 should be added between the two integers.5 Segment.

6.4.1.2Intramuscular fat determination

A) Determination time: it is advisable to stop breathing within 1 h to 2 h. If the measurement is delayed, the water loss and deterioration of meat samples should be avoided:

Bb) measured site: the longest dorsal muscle in the lumbar spine:

C) Sample preparation: except for the peripheral fascia, cut into small pieces in the meat grinder and twisted into minced meat for testing:

And d) weigh 10.0000g ± 0.0500g with a balance and record it:

E) Place the minced meat in a wide bottle, add 60 mL of methanol, cover the bottle cap, and stir it on a magnetic mixer for 30 min:

F) Open the cap and add 90 mL of trichloromethane. Cover the cap, stir until the minced meat is flocculate and suspended in the solvent. Leave for 36h

Should be shaken 3~4 times:

G) The extract was filtered into the graduated fraction funnel and the residue was washed with about 50 mL trichloromethane:

H) The funnel was removed, 30 mL of distilled water was added, the split liquid funnel was static and layered, the upper layer is water methanol layer, and the lower layer is trichloromethane lipid

Level, record the lower layer volume. After slowly opening the split funnel valve and discard about 2 mL, slowly release the lower fluid into the beaker: i) take 4 clean beakers and dry, weigh, and record the weight of the beaker:

J) Remove the 50.00mL lower liquid out of the heavy beaker with a pipette, place and dry the liquid on the electric heating plate, then place the beaker in the oven, bake at 105℃ ± 2℃ for 1h, cool the beaker in the dryer to room temperature, weigh and record it:

K) Make two parallel samples for each meat sample, and the relative deviation between the two parallel samples shall be less than 10%, otherwise it shall be redone.

6.4.2 Description of the measurement results

6.4.2.1The marble score results are expressed as in formula (9):

$$\text{Marbling pattern} = \text{visual assessment score:} \quad (9)$$

6.4.2.2The results of intramuscular fat determination are expressed as in equation (10):

$$\text{Intramuscular fat (\%)} = [(W_2 - W_1) / (W_0 \ 50 / V_1)] 100 \quad (10)$$

In formula:

W_0 —— meat sample weight, the unit is in grams (g):

W_1 —— Beaker weight, the unit is in grams (g):

W_2 —— Beaker with fat weight, measured in grams (g):

V_1 —— Total volume of lower fluid in milliliter (mL):

50 —— sample volume in ml (mL).

6.4.2.3The results were expressed as means and retained to two decimal places.

6.5 Moisture

6.5.1 Determination method

6.5.1.1Determination time, determination site and sample preparation are the same6.4.1.2.

6.5.1.2Take 2 clean weighing bottles and dry, weigh and record the weight of the bottles.

6.5.1.3Take the minced meat in the weighing bottle, make the minced meat occupy 2 / 3 of the weighing bottle, flatten and weigh it as the weight before drying.

6.5.1.4Place the weighing bottle in vacuum drying, open the weighing bottle cap by 1 / 3, and bake at 60℃~65℃,0.25Mpa ~ 0.20 Mpa for 24h.

6.5.1.5Heat up to 102℃ ± 2℃, bake at 0.25 Mpa ~ 0.20 Mpa for 2h, remove it in a dryer to cool to room temperature and weigh. Repeat until the constant weight (the difference between the two weights is less than 0.01g).

6.5.1.6Each meat sample measures two parallel samples, and the relative deviation between the results of the two parallel samples shall be less than 5%, otherwise it shall be redone.

6.5.2 Description of the measurement results

The test results are expressed as in equation (11):

$$\text{Muscle moisture (\%)} = [(W_1 - W_2) / (W_1 - W_0)] 100 \quad (11)$$

In formula:

W_0 —— weighing bottle weight in grams (g):

W_1 —— weighs the sum of the bottle and meat sample in grams (g):

W_2 —— weighs the sum of the bottle and meat sample in grams (g).

Asresults are expressed as means and retained to two decimal places.

7 Pig muscle quality determination

7.1 Principles of determination

The determination of pig muscle quality is based on meat color, pH and hydraulic force, and other indicators as reference.

7.2, normal meat

7.2.1 Color value

10% to 25%: or flesh color score of 3 to 4.

7.2.2 pH

PH_1 : 5.9~6.5 or PH_{24} : 5.6~6.0.

7.2.3 Dripping loss

2%~6%, or water loss rate of 6%~15%, or tie the 80%~95% hydraulic force.

7.3 PSE meat

7.3.1 Color value of 26%, or a flesh color score of 1 to 2 points.

7.3.2 pH_1 5.9, or pH_{24} <5.5.

7.3.3 Dripping loss> 6.1%, or water loss rate> 15.1%, or tie hydraulic less than 80%,

7.4 DFD meat

7.4.1 Color value of <10%, or flesh color score of 5 to 6.

7.4.2 pH_1 > 6.5, or pH_{24} > 6.0.

7.4.3 Dripping loss <2%, or water loss rate <5%, or tie water force> 95%.

7.5 Acid meat (RN)

When pH_1 is above 6.1 and pH_{24} is below 5.5, the muscle can determine acid meat (RN)..

6

The "New Media Matrix" of the State Administration for Market Regulation

Official wechat of the State Administration of Market Regulation

Official Weibo account of the State Administration for Market Regulation

State Administration for Market Regulation official TikTok

State Administration of Market Regulation official fast hand

* People's, The Paper, netease, Toutiao, Baijia, Little Information, Big Fish, Tencent Penguin, you can search "Xinyu" on the client and web pages of relevant platforms.

All copyright infringement must be prosecuted

Supervisor: The Standardization Administration of China
Host: The National Standard and Technical Evaluation Center of the State Administration for Market Regulation
Technical support: Beijing Bid-Saiyu Technology Co., LTD
Support telephone number: 13261900266

friendly link

The Standardization Administration of China

Enterprise standard information public service platform

National group standard information platform
